
JOURNAL INFORMATION
==============================
NLM Title Abbreviation: Atlanta Med Surg J (1884)
NLM Journal ID: 101251623

Atlanta Medical and Surgical Journal (1884)

Publisher: Biomedical Journal Digitization Project

ARTICLE INFORMATION
==============================
PMCID: PMC9199298
Article version: 1
Subjects: Book Reviews, Pamphlets, Exchanges

Book Reviews

Print publication date: 1898 Dec
Volume: 15
Volume Series: New Ser
Issue: 10
First page: 699
Last page: 704
License: This work is free of known copyright restrictions. For more information, please see PMC Back Issue Digitization.
License URL: https://creativecommons.org/publicdomain/mark/1.0/

==============================

### BOOK REVIEWS, PAMPHLETS, EXCHANGES.

### BOOK REVIEWS.

[Contributions solicited for review.]

A Clinical Text-Book of Medical Diagnosis for Physicians and Students. By Oswald Vierordt, M.D., Professor of Medicine at the University of Heidelberg. English translation by Francis H. Stuart, M.D., of Brooklyn, N. Y. Published by W. B. Saunders, Philadelphia. Price, Cloth, $4.00; Sheep, $5.00.

This excellent text-book on physical diagnosis has just been issued from the well-known publishing house of W. B. Saunders. It is the fourth American from the fifth German edition. Its first edition was issued in 1888, and in ten years four others have been called for. This attests the merits of the book, when we consider the number of excellent works on this same subject. The book is a large one and treats very exhaustively the diagnosis of every disease of the body where physical signs are considered. Some idea may be obtained as to the scope of the work from arrangement of its contents.

There are chapters as follows:

Chapter I. Introduction.
Chapter II. Examination of Patients.
Chapter III. General Examination.
Chapter IV. Examination of the Respiratory Apparatus.
Chapter V. Examination of the Circulatory Apparatus.
Chapter VI. Examination of the Digestive Apparatus.
Chapter VII. Examination of the Urinary Apparatus.
Chapter VIII. Examination of the Nervous System.

The last chapter on nervous diseases is unusually clear and thorough, and is in itself the value of the whole work. The methods for diagnosis, as laid out so explicitly, are quite refreshing. We commend most thoroughly this work of Dr. Vierordt's.

A MANUAL OF VENEREAL DISEASES. By James R. Hayden, M.D., Chief of Clinic and Instructor in Genito-Urinary and Venereal Diseases, College of Physicians and Surgeons, New York; Professor of Genito-Urinary and Venereal Diseases in the Medical Department of the University of Vermont, etc. New (2d) edition, revised and enlarged. In one 12mo volume of 304 pages, with 54 engravings. Cloth, $1.50 *net.* Lea Brothers & Co., Publishers, Philadelphia and New York.

Though a manual, and hence condensed, the subjects treated are quite fully gone into. While acknowledging Neisser's gonococcus as the cause of the majority of cases of gonorrhea, he lays stress upon the fact that many, indistinguishable from the genuine, cases are due to infection with "innocent" secretions. He appears not to favor the Janet method of treatment. It is not yet proven, he says, that Ducrey's bacillus is the cause of chancroid. Many "chancroids" are simple, non-specific, infective ulcers.

Syphilis, in all its stages, forms and locations, is very thoroughly, though concisely described; every organ and tissue which the disease may affect being mentioned, with the symptoms as displayed by the part involved.

He advises no mercurial treatment until the disease is thoroughly established, and considers inunctions the most efficacious. The intermittent plan of treatment is followed. The "mixed" treatment is begun with the second year. As iodides only remove symptoms, mercury is not left off in the late stages.

The book ends with the discussion of hereditary syphilis.

M. B. H.

---

PATHOLOGY AND MORBID ANATOMY. By T. Henry Green, M.D., Lecturer on Pathology and Morbid Anatomy at Charing-Cross Hospital Medical School, London. New (8th) American from the eighth and revised English edition. In one very handsome royal octavo volume of 600 pages, with 215 engravings, many being new, and a colored plate. Cloth, $2.50 *net.* Lea Brothers & Co., Publishers, Philadelphia and New York.

This is the eighth edition of this work. That speaks for itself. It has probably been the most popular text-book on pathology and morbid anatomy that has ever been published. The reason for this is patent when the volume has once been thoroughly examined. The arrangement and classification of the subjects are most excellent. Ten years ago the reviewer of this book studied it as a text-book at the University of Virginia, and in looking over it now in this edition, experience and study aptly show the value of the book. The language is clear and concise and the illustrations are good We shall always commend this work.

---

A PRIMER OF PSYCHOLOGY AND MENTAL DISEASE, FOR USE IN TRAINING-SCHOOLS FOR ATTENDANTS AND NURSES AND IN MEDICAL CLASSES. By C. B. Burr, M.D., Medical Director of Oak Grove Hospital for Nervous and Mental Diseases, Flint, Mich.; formerly Medical Superintendent of the Eastern Michigan Asylum; Member of the American Medico-Psychological Association, etc. Second edition, thoroughly revised. 5½ x 7¾ inches. Pages ix–116. Extra cloth, $1.00 net. The F. A. Davis Co., Publishers, 1914–16 Cherry St., Philadelphia; 117 W. Forty-second St., New York City; 9 Lakeside Building, 218–220 S. Clark St., Chicago, Ill.

While this little book is a primer, yet it deals with subjects so intricate that even its pages must be read by those whose mind is given to much thought. In the first edition of this work its author dealt with psychology and were lectures delivered to the Training-school Classes in Eastern Michigan Asylum, but in this edition a little more has been incorporated. Part I. deals with Psychology, which is very clear and, as was meant, rudimentary in character. Part II. treats of Insanity, and Part III. treats of the Management of Cases of Insanity, especially for nurses and is well worthy of consideration by physicians. Part IV. is a lecture delivered to Training-school Class, and is "Suggestions as to What to Do and What to Avoid in Caring for the Insane."

---

LANDIS' COMPEND OF OBSTETRICS. Revised and edited by Dr. Wm. H. Wells, of Philadelphia. Published by P. Blakiston's Son & Co., of Philadelphia. Price 80 cents.

It is almost needless to refer to this excellent little compend of obstetrics which has already reached its sixth edition. It is thorough and practical throughout, and also contains some good illustrations. Of course, it is nothing but a "quiz compend," and therefore, intended to aid students in preparing for examinations. There can be no doubt but that such a book well takes the place of a "quiz master."

---

A TREATISE ON THE SCIENCE AND PRACTICE OF MIDWIFERY. By W. S. Playfair, M.D., LL.D., F.R.C.P., Emeritus Professor of Obstetric Medicine in King's College, London. Examiner in Midwifery to the Universities of Cambridge and London. Seventh American from the ninth English edition. In one very handsome octavo volume of 700 pages, with 207 engravings and seven full page plates. Cloth, $3.75 *net*. Lea Brothers & Co., Publishers, Philadelphia and New York.

It is hardly necessary to review such a well-known work as this, which has already reached its seventh American and ninth English edition. It has been a standard work on obstetrics for years, and an exceedingly popular text-book in medical colleges, and we are sure that the present edition will meet with the same general favor. The language used is exceedingly clear and explicit, which accounts in a large measure for its popularity among medical students. The arrangement is good, and it must still continue to hold a very high place as a text-book.

---

TREATMENT OF SKIN CANCERS. By W. S. Gottheil, M.D., Professor of Dermatology at the New York School of Clinical Medicine; Dermatologist to the Lebanon Hospital, the North-Western and West-Side German Dispensaries, Etc. Published by *The International Journal of Surgery Company*, 100 William Street, New York. Price $1.00.

This is a very small book, giving in brief, a general consideration of the subject, but chiefly devoted to the diagnosis and treatment. The illustrations are good. The little book is extremely interesting. In the treatment he advocates, for most cases, curetting and Marsden's Paste, following much the line of A. R. Robinson. Whether one is ready to adopt the author's views in full or not, the work is one which is well worth having. His opinion of the treatment is that held by quite a number of dermatologists, as opposed to the surgeons' preference. M. B. H.

MANUAL OF DISEASES OF THE SKIN, with an Analysis of Twenty Thousand Consecutive Cases, and a Formulary. By L. Duncan Bulkley, A.M., M.D., Physician to the New York Skin and Cancer Hospital; Dermatologist to the Randall's Island Hospital, etc. Fourth edition, revised and enlarged. G. P. Putnam's Sons, New York and London. 1898.

This might justly be called a primer. on diseases of the skin. Though brief, it is from the pen of one who has had years of large clinical experience, and knows well his subject. The book could hardly be called as fully up-to-date as some others, but this does not necessarily depreciate it. There are no illustrations. General considerations and a formulary complete the work. The management of the subjects is one which will appeal forcibly to the general practitioner. M. B. H.

---

THE MEDICAL NEWS VISITING LIST FOR 1899. Weekly (dated, for 30 patients); Monthly (undated, for 120 patients per month); Perpetual (undated, for 30 patients weekly per year); and Perpetual (undated, for 60 patients weekly per year). The first three styles contain 32 pages of data and 160 pages of blanks. The 60-patient Perpetual consists of 256 pages of blanks. Each style in one wallet-shaped book, with pocket, pencil and rubber. Seal Grain Leather, $1.25. Thumb-letter Index, 25 cents extra. Philadelphia and New York: Lea Brothers & Co.

Messrs. Lea Brothers have again presented us with a complimentary copy of their "Visiting List for 1899." Like all former editions the arrangements are excellent.

---

A POCKET MEDICAL DICTIONARY. By Geo. M. Gould, A.M., M.D., of Philadelphia. A new edition. Published by P. Blakiston's Son & Co., Philadelphia. Price $1.00.

This is a Pocket Medical Dictionary, thoroughly correct, handy and contains over 21,000 words. There are others, but this is the original, for Dr. Gould was the first lexicographer who compiled such a useful little book, and which has met with such unprecedented success. This last edition has been entirely revised and will doubtless be more popular than ever.

AMERICAN POCKET MEDICAL DICTIONARY OF 26,000 WORDS. By W. H. Newman Dorland, A.M., M.D. Published by W. B. Saunders. Price, $1.25 net.

This is another pocket edition dictionary. It is excellent and beautifully gotten up. The binding and the paper are excellent, and is a work to be commended.

---

SAUNDERS' QUESTION COMPENDS—ESSENTIALS OF MATERIA MEDICA AND THERAPEUTICS. By Henry Morris, M.D.

This is the fifth revised edition of this well-known compend. Saunders' Question Compends have already shown their merit and therefore need no further introduction.

---

THE PHYSICIAN'S VISITING LIST FOR 1899. Forty-eighth year of its publication. Published by P. Blakiston's Son & Co., Philadelphia.

The Visiting List for 1899 is up to the usual standard of this well-known house. The covering is exceedingly durable and the paper excellent, besides the form is very handy.

---

### REPRINTS RECEIVED.

Hydrotherapy in the Treatment of Nervous and Mental Diseases. By Frederick Peterson, M.D., New York.

Discussion on Hydrotherapy before the German Medical Society of New York.

Manifestations of Syphilis in the Mouth.

The Dangers of Specialism in Medicine. By L. Duncan Bulkley, A.M., M.D., New York.

Deaths (Ten); Surgical and Causes.

The Dermal Coverings of Animals and Plants.

Serpents and their Venom: Copperhead, Coral and Rattlesnake. By B. Merrill Ricketts, Ph.B., M.D., of Cincinnati.

The Surgical Treatment of Uterine Myomata. By H. O'Marcy, A.M., M.D., Boston.

Journal of Proceedings of the National Pure Food and Drug Congress, held in Washington, D. C., March 2, 3, 4, and 5, 1898.
